# A potential prognostic marker for hematologic neoplasms: CD58

**DOI:** 10.3389/fonc.2025.1586842

**Published:** 2025-04-29

**Authors:** Jiajia Cao, Yurong Zhang, Ningning Yue, Shuzhen Xiong, Shuni Zhang, Chongyang Wu

**Affiliations:** Department of Hematology, The Second Hospital of Lanzhou University, Lanzhou, China

**Keywords:** biomarker, CD58 antigens, CD2 antigens, immunological synapses, hematologic neoplasms

## Abstract

CD58 is a glycoprotein receptor widely distributed on histiocytes that binds to CD2, that takes part in constituting the Immunological Synapses (IS) and activating T/NK cells. Aberrant expression of CD58 has been demonstrated to exert a significant impact on the prognosis of hematological tumors, including leukemia and lymphoma. Furthermore, this aberrant expression has been associated with drug resistance and immune rejection in CAR cell therapy. In this article, we will explore the future of CD58 in hematological oncology by describing its function in immune cells, its impact on hematological oncology and immunotherapies such as CAR cell therapy.

## 1 Introduction

CD58 antigen is a glycoprotein receptor that is widely expressed on dendritic cells, T cells, NK cells, and other types of tissue cells. It has a molecular weight of 40–70 kDa and a high level of glycosylation (1). It plays a pivotal role in the immune response by binding to CD2 molecules on the surface of immune cells, participating in the constitution of Immunological Synapses (IS), and activating T cells and NK cells (2).CD58 antigen not only participates in the interaction and signaling between immune cells, but it is also closely related to the development of immune-related diseases, such as tumors, which ultimately affect the prognosis of the disease.

Previous studies have demonstrated that CD58 exhibits a dual role in either promoting or inhibiting tumor growth, depending on the specific tumor type. For instance, elevated CD58 expression in gliomas has been proposed as a potential immunosuppressive factor, contributing to a worse prognosis (3–5). Conversely, high expression of CD58 in hepatocellular carcinoma and colorectal carcinoma has been linked to tumor development through the upregulation of the Wnt pathway (6, 7). However, in melanoma and ductal A/B breast cancer, CD58 functions as an anti-tumor effector. High expression of CD58 has been shown to predict a more favorable prognosis in melanoma and ductal A/B breast cancer (8–10). In hematologic tumors, CD58 is frequently present as an antitumor effector, and CD58 deficiency is associated with a poor prognosis in leukemia and lymphoma (11).

Furthermore, the absence of CD58 expression has been linked to resistance against CAR cell therapy (12). Hammer et al. demonstrated that knocking down adhesion molecules, specifically CD58, on T/NK cells can effectively eliminate host NK cell-mediated rejection. This finding presents a new strategy for solving the challenge of immune rejection from CAR products, particularly CAR-NK cells (13).

All of these suggest that the CD58 antigen has excellent potential to help treat hematologic cancers. In this paper, we will deeply describe the function of CD58 in immune cells, its impact on hematological tumors, and its role in CAR cell therapy and other immunotherapies.

## 2 Structure and isoforms of CD58

**Figure 1:**
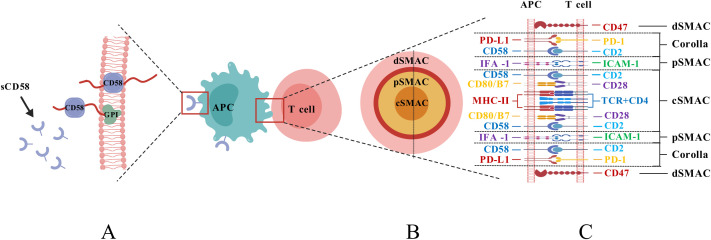
**(A)** Structural diagram of the isoforms of CD58; **(B)** Structural diagram of the interface of the IS; **(C)** Composition of the IS structure.).

The surface glycoprotein CD58, often called lymphocyte functional antigen 3 (LFA-3), is highly glycosylated (40–70 kDa) and abundantly expressed on different histiocytes such as dendritic cells (DC), and it functions by binding to CD2 on the outer layer of T/NK cells (11). Its two isoforms are glycosylphosphatidylinositol (GPI)-anchored form and type-I transmembrane form (**Figure 1A**), the GPI-anchored isoform is found in lipid rafts. It is crucial for promoting cell adhesion and creating stable IS. In contrast, the transmembrane form is found in structural domains outside lipid rafts and activates signaling pathways like AKT, ERK, Wnt/β-catenin, and GSK3β (6, 14). The extracellular region of the GPI-anchored isoform comprises two immunoglobulin-like structural domains. The membrane distal structural domain connects to T11.1 and T11.2 of CD2, and the membrane-proximal structural domain helps CD58 stick to the cell. The transmembrane type possesses a singular extracellular structural domain (15). It has also been found that there is a soluble form of CD58 that is thought to come from the splitting of GPI-anchored forms. This soluble form might compete with CD58 for binding, which could mess up antigen-presenting systems and be linked to tumor immune evasion and resistance to immunotherapy (16, 17).

## 3 CD58 effect on immune cells

### 3.1 Effects on T cell activation and apoptosis

#### 3.1.1 CD58 plays a critical role in forming the IS

Following the recognition of T cells and antigen-presenting cells (APCs), the structure that emerges at the contact interface is characterized by cytoskeletal remodeling and dynamic rearrangement of receptor-ligand interactions, referred to as the Immunological Synapses (IS) (18). This structure is composed of supramolecular activation clusters (SMACs), which include molecules such as T Cell Receptor (TCR), MHC molecules, CD2, CD28, and CD58. The IS is divided into distinct regions: the central SMAC (cSMAC), peripheral SMAC (pSMAC), and distal SMAC (dSMAC), collectively forming a pattern resembling a bull’s eye at the cell contact interface (19, 20) (**Figure 1B, C**). The CD2-CD58 complex cluster is a crucial element of the IS, present in the cSMAC and forming a “corolla”-like structure that sits between the dSMAC and the pSMAC (11, 20). The corolla incorporates co-stimulatory molecules like CD28, ICOS, CD226, and SLAM-F1, augmenting phosphorylated Src family kinase (pSFK), and phospholipase C-γ (PLC-γ). The CD2-CD58 complex cluster in the corolla promotes signaling by 77% relative to the CD2-CD58 complex cluster in cSMAC (20). Furthermore, the distance after CD2-CD58 contact between T cells and APCs was around 135 μm, closer to the distance necessary for TCR binding to MHC, suggesting that the binding of CD58 to CD2 is more favorable for TCR signaling from a physical distance (21).

#### 3.1.2 CD58 delivers co-stimulatory signals

**Figure 2:**
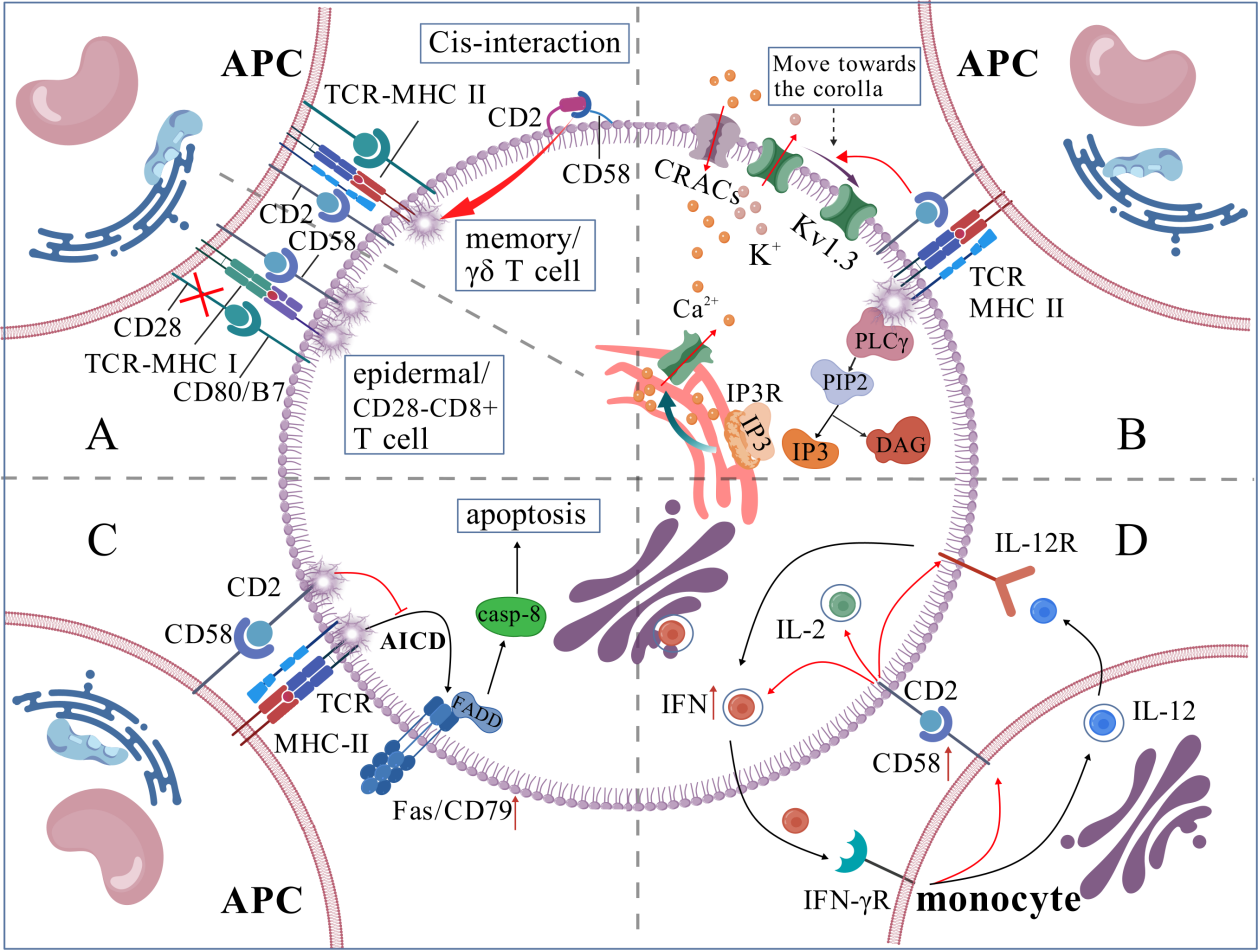
**(A)** CD58 is involved in T cell activation process; **(B)** CD58 regulates intracellular Ca^2+^ levels; **(C)** CD58 inhibits T cell apoptosis; **(D)** CD58/CD2 plays a central functional connection in IL-12/IFN-γ positive feedback loop.).

CD58 participates in T cell activation by providing co-stimulatory signals to T cells (11). CD28^-^CD8^+^ T cells, prevalent in prolonged chronic infections and tumor microenvironments, lack co-stimulatory signals via the CD28-B7 pathway; hence, CD2-CD58 is the primary co-stimulatory signaling route (22). In Cytomegalovirus seropositive People With HIV cells, CD2 expression on CX3CR1^+^CD57^+^CD28^-^CD8^+^ T cells was elevated, augmenting TCR-mediated activation of inflammatory CD8^+^ memory T cells via CD2-CD58 co-stimulation (23). CD57^+^CD4^+^ T cells, indicative of senescent T cells, exhibited high expression levels of CD2, making CD2-CD58 the most critical co-stimulatory molecule in these cells (24). Most epidermal T cells lack CD28 expression but express co-stimulatory markers, including CD2 and CD6; hence, the CD2-CD58 axis plays a significant role in the epidermal immune response (25, 26) (shown in **Figure 2A**). Certain tumor-infiltrating T cells exhibit elevated PD-1 expression, and the interaction between PD-1 and PD-L1 can negate CD28 co-stimulation, thereby diminishing T cell responses, however, CD2-CD58 co-stimulatory signaling demonstrates reduced sensitivity to PD-1 inhibition, establishing it as a crucial co-stimulation-adhesion axis that facilitates an anti-tumor response (27). A dual blockage technique with Siplizumab (A humanized monoclonal antibody targeting CD2)and Abatacept/Belatacept was necessary to entirely inhibit the activation of allogeneic reactive CD4^+^/CD8^+^ T cells, it also indicates the significance of the CD2-CD58 co-stimulatory axis in T cell activation (28). Moreover, the CD2-CD58 axis, while not an indispensable co-stimulatory signal for T cell activation, amplifies TCR signaling (29), and cell adhesion induced by CD2-CD58 contributes to the sensitivity of TCR recognition, particularly in memory T cells exhibiting elevated CD58 expression (30, 31). Furthermore, Li et al. discovered that T cell activation necessitates the cis contact of CD2 with CD48/CD58, a function that is particularly significant in memory cells and innate-like T cells (NK-T cells, γδ T cells) (**Figure 2A**) (31).

#### 3.1.3 CD58 facilitates the generation and stability of calcium signals

The activation of T-cells depends on calcium signal strength and frequency. TCR-MHC activates PLC-γ, which breaks down phosphatidylinositol-4,5-bisphosphate (PIP2) into Inositol 1,4,5-Trisphosphate (IP3) and diacylglycerol (DAG). IP3 binds to its receptor on the endoplasmic reticulum (ER) membrane, releasing Ca^2+^ from the ER lumen. This initial Ca^2+^ release is minor and insufficient for gene activation. When ER Ca^2+^ levels drop, calcium release-activated channels are activated, allowing substantial Ca^2+^ influx from the extracellular environment. If sustained, this Ca^2+^ influx activates T cells. When T cells are exposed to extracellular calcium ions, the potassium channel Kv1.3, which is present in significant concentrations within human T cells, relocates from the F-actin-dense distal compartment of the synaptic interface to the IS. This movement helps maintain the negative membrane potential needed for Ca^2+^ entry. The CD2-CD58 corolla structure facilitates the formation of the distal loop of Kv1.3 and sustains Ca^2+^ signaling (19) (**Figure 2B**).

#### 3.1.4 CD58 prevents T-cell apoptosis

IL-2 enhances T-cell proliferation and differentiation and facilitates survival (32), whereas the CD2-CD58 interaction stimulates IL-2 secretion via the NF-κB pathway (33). Moreover, CD58 on DC might impede the TCR activation-induced apoptotic process of T cells by obstructing CD3-mediated Fas/Fas-L overexpression (34) (**Figure 2C**). The stimulatory signals produced by the interaction of CD58 on active monocytes with CD2 on CD56^+^ T cells are crucial for the growth and differentiation of these T cells (35). Moreover, the majority of the co-stimulatory pathways that deplete T cells (like CD28-B7, CD40-CD40L, etc.) may have dropped their capacity to activate T cells effectively; however, the CD2-CD58 pathway continues to preserve its stimulatory activation function, presenting significant potential for reversing T cell unresponsiveness and mitigating T cell depletion (15).

### 3.2 CD58 participates in NK cell-mediated cytotoxicity

Analogous to its function on T cells, CD58 contributes to the formation of the IS (NKIS) together with CD2 on NK cells, where the CD2-CD58 complex is situated at the periphery of the NKIS and delivers a crucial secondary signal for NK cell activation (9, 36). In the CD56^dim^ subpopulation, CD58 facilitates the localization of CD2 to the NKIS, enabling CD2 at the NKIS to recruit CD16 to the NKIS (15). This interaction benefits the binding of CD16 to the Fc regions of antibodies targeting the cells, thereby mediating cytotoxic effects, conversely, the absence of CD2-CD58 axis signaling markedly diminishes NK cell cytotoxicity without impacting Antibody-Dependent Cell-Mediated Cytotoxicity (ADCC) effects (37). The CD56^bright^ subset, which has strong CD2 expression, facilitates cytokine production, including IFN-γ and TNF-α, through the interaction of CD58 and CD2 (9). A genome-wide CRISPR screen identified CD58 as significantly enriched in NK cell genes, and its deletion resulted in diminished NK cell cytotoxicity (36). Further co-culture data indicated that the deletion of CD58 in melanoma cells gave resistance to cytotoxicity mediated by T cells and NK cells (8). In Nalm 6 cells, a human B-lymphoblastic leukemia cell line, the deletion of CD58 enables these cells to resist destruction by NK cells (38). Interestingly, NK cells also express CD58, Schwane et al. discovered that it is predominantly found in CD56^dim^ subset, exhibiting significant interindividual variability in expression (39). The functional implications of CD58 in NK cells remain largely unexplored, suggesting a potentially intricate role. Moreover, in CAR cell therapy, CD58 on CAR-T/NK cells functions as an adhesion ligand, and takes part in host T/NK cell-mediated immunological rejection (13).

### 3.3 Role of other immune cells

The interaction of CD58 molecules on DC with CD2 on T cells is essential for initiating adaptive immune responses (8). Exposure to parasites results in increased expression of the adhesion protein CD58 on the surface of Monocyte-Derived DC (40). Th1 cells secrete more IFN-γ when CD58 on monocytes and CD2 on T cells connect, which also starts the best possible T-cell response to IL-12 (41). Additionally, the binding of periplasmic IFN-γ to IFN-γR on monocytes stimulates monocytes product IL-12 and the expression of CD58. Monocyte-derived IL-12 subsequently induces the secretion of cytokines IL-2 and IFN-γ in Th1 cells, thus generating a positive feedback cycle between monocytes and T cells via IL-12/IFN-γ. This process is facilitated by the CD2/CD58 interaction, which influences physical proximity, promotes IFN-γ secretion, and enhances T-cell sensitivity to IL-12 (42, 43) (**Figure 2D**). Furthermore, Yamamoto et al. discovered that CD58 expression on monocytes may suppress the immunological function of regulatory T cells and heighten susceptibility to autoimmune thyroid disease when CD58 expression is diminished (44). The expression of CD58 on B lymphocytes fluctuates according to various physiological and pathological conditions. Initially, Bone marrow cells exhibit significant levels of CD58 expression on their surface; however, as B cells undergo differentiation, CD58 expression progressively diminishes (11). CD58 expression is usually upregulated in Acute Lymphocytic Leukemia (ALL) cells: CD58 expression is typically upregulated and markedly elevated in ALL progenitor cells compared to normal B cells (45). The subsequent table delineates the expression and role of CD58 across different cell types (**Table 1**)

**Table 1:** Expression and function of CD58 in various cells.

Cell	Expression	Function	Ref
CD4^+^/CD8^+^ T Cells	+	Participate in the immune rejection between CAR-T cells and hosts; CD2 interact with CD58 on target cells forms IS, promotes T cell activation, and inhibits apoptosis.	(22, 46)
Memory T Cells	+	Involve in T cell activation by cis interaction with CD2; Promote the secretion of cytokines such as IFN-y.	(31)
Regulatory T Cells	NA	CD58 on monocytes inhibits the function of regulatory T cells.	(44)
γδT Cells	+	Involve in T cell activation by cis interaction with CD2.	(31)
NK Cells	+	Participate in the immune rejection between CAR-NK cells and hosts; CD58 on target cells interacts with CD2 on NK cells:form NKIS, provide co-stimulatory signals, and promote the secretion of cytokines.	(15, 36)
Dendritic cells	+	Involve the formation of IS; provide co-stimulatory signals for T cell activation.	(19, 22)
Monocyte	+	Increase T cell response to IL-12 and promotes the formation of IL-12/IFN-y positive feedback loops.	(11, 42)
B cells	+	NA	(11)
Thymic epithelial cells	+	Bind to CD2 on thymocytes: involve in thymocyte proliferation, selection, maturation, and activation.	(11, 15)
Keratinocytes	+	Interact with CD2 on naive T cells to promote their activation and Th1 differentiation, which is associated with the development of psoriasis	(26)
Endothelial cells	+	Interact with CD2 on T cells to enhance their recruitment, activation, and increase the permeability of endothelial cells, this is involved in inflammatory processes.	(47)
Intestinal epithelial cells	+	Interact with CD2 on T cells to promote T cell activation and cytokine secretion. This is involved in intestinal immune processes.	(11)
Neuroglia	+	Involve in antigen recognition and presentation.	(48)
Peritoneal mesenchymal stromal cells	+	NA	(49)
Choroidal neuronal cells	–	NA	(50)

“+” represents the presence of CD58 protein expression on the cell membrane, while “-” indicates the absence of CD58 protein expression on the cell membrane. NA stands for no current research.

## 4 Role of CD58 in hematologic neoplasms

### 4.1 Leukemia

#### 4.1.1 B-cell precursor acute lymphoblastic leukemia (BCP-ALL)

Lymphoblastoid cells exhibit elevated CD58 expression and diminished CD81 expression relative to normal hematopoietic cells, allowing the CD81/CD58 expression ratio to serve as a diagnostic tool for distinguishing between hematopoietic and lymphoblastoid cells in BCP-ALL cases (45). Hyperdiploid BCP-ALL exhibits markedly elevated CD58 antigen expression compared to other haplotypes, potentially elucidating, albeit to a minor degree, the superior prognosis of hyperdiploid patients (51). The Minimal Residual Disease (MRD) test is the primary prognostic indicator for BCP-ALL. It has been noted that CD58 is overexpressed in over 90% of cases, thereby enhancing MRD detection alongside marker molecules such as CD19, CD22, and CD24 (52). This methodology has been implemented in the United States to identify BCP-ALL MRD (53). Nonetheless, CD58 expression in leukemia cells may be markedly down-regulated following induction chemotherapy (54), so this issue needs to be noted when employing CD58 for MRD prognostic monitoring.

#### 4.1.2 Acute B-lymphoblastic leukemia (B-ALL)

A cohort study by Diamanti et al. indicated that CD58 was overexpressed in as many as 75% of ALL patients (55), and is associated with a poorer prognosis (56). Conversely, a cohort study involving pediatric and young adult B-ALL indicated that high expression of CD58, along with elevated levels of CD123 and CD81, correlated with extended progression-free survival, particularly noting significant overexpression of CD58 in cases with recurrent cytogenetic abnormalities and t (1:19) (45). Overall, B-ALL with KMT2A mutations is associated with a poorer prognosis, a study examining tumor cell surface antigen expression in patients with KMT2A mutations revealed that KMT2A Ar^+^ cells exhibited heightened expression of CD45, CD38, and CD58 (57), indicating that CD58 may serve as a significant prognostic marker for B-ALL. In relapsed/refractory B-ALL, treatment typically necessitates bispecific antibodies or CAR-T cell therapy; however, induction agents like vincristine and prednisone often down-regulate CD58 expression on tumor cells, consequently reducing the effectiveness of Blinatumomab and anti-CD19^-^CAR-T in B-ALL (12, 58, 59). The discovery that the CD2-CD58 axis inhibits Blinatumomab-induced T-cell activation in its absence is a primary factor contributing to Blinatumomab resistance in patients with intact CD19 expression. This insight enables personalized treatment strategies for patients exhibiting defective CD58 expression or those with PAX5 P80R, potentially guiding the decision to forgo Blinatumomab therapy (58, 60). This finding also implies considerations for the clinical management of high-risk patients with unfavorable prognoses: For clinically initially treated high-risk B-ALL patients at great risk of relapse and patients who are willing to intervene early with CAR-T cell therapy, it is important to take into account the need for later CAR-T cell therapy or Blinatumomab to minimize the use of cytotoxic drugs such as Zoerythromycin, Prednisolone, or Vincristine and to shorten the duration of bridging therapy to avoid the downregulation of CD58 in order to ensure the efficacy. Nonetheless, extensive samples are required to confirm the impact of chemotherapeutic agents on the up-or-down-regulation of CD58 and investigate the dose dependence of these effects and the minimal threshold required for their performance.

#### 4.1.3 Acute myeloid leukemia (AML)

In AML, CD58 expression is positively associated with the rates of Disease-Free Survival (DFS), Complete Response (CR), and Overall Survival (OS). Defective CD58 expression in Chronic Myeloid Leukemia (CML) progenitors impedes the normal proliferative response of T lymphocytes, resulting in abnormal adherence and clonal proliferation of CML progenitor cells, hence contributing to immune evasion (11). Chen et al. discovered that diminished CD58 expression correlates with unfavorable OS in cytogenetically normal acute myeloid leukemia (CN-AML) patients and suggested a combined WT1 and CD58 model for prognostic risk evaluation in CN-AML patients, which complements existing prognostic assessment methodologies for CN-AML (61).

### 4.2 Lymphoma

#### 4.2.1 Hodgkin’s lymphoma (HL)

HL patients exhibit elevated levels of CD58, which interacts with CD2 on CD4^+^ T cells to create a “rosette” structure, this phenomenon is linked to the CD58-mediated swift and extensive re-localization of CD2 near the IS formation site, accompanied by significant F-actin polymerization, the reduction of CD58 expression or blockade of CD2 diminishes wreath formation and T cell activation (29, 62). Mutations of CD58 in primary Hodgkin Reed-Sternberg (HRS) cells are infrequent; however, pure or heterozygous inactivating mutations of CD58 are prevalent in HL cell lines. These mutant genes typically result in exon three deletions or other aberrant RNAs or may produce no RNAs whatsoever. Furthermore, these aberrant RNAs do not express CD58. The inactivation of CD58 is notably common in HRS cells found in pleural effusions during the advanced stages of HL (63). CD58 expression is elevated in most HRSdx cells that exhibit resistance to multiple treatments, including vincristine, gemcitabine, and bendamustine hydrochloride (64).HR cells exhibit elevated expression of CD58, and a substantial population of activated, rosette-like CD4^+^ T cells marks the microenvironment. Nevertheless, diverse inhibitory signaling molecules and tumor escape-related signaling molecules render T cells non-responsive; moreover, forming a rosette with CD58 establishes a physical barrier that obstructs CD8^+^ T cell and NK cell-mediated killing (29, 65), Thus, high expression of CD58 by HL cells is associated with the promotion of tumorigenesis and development.

#### 4.2.2 Diffuse large B-cell lymphoma (DLBCL)

**Figure 3:**
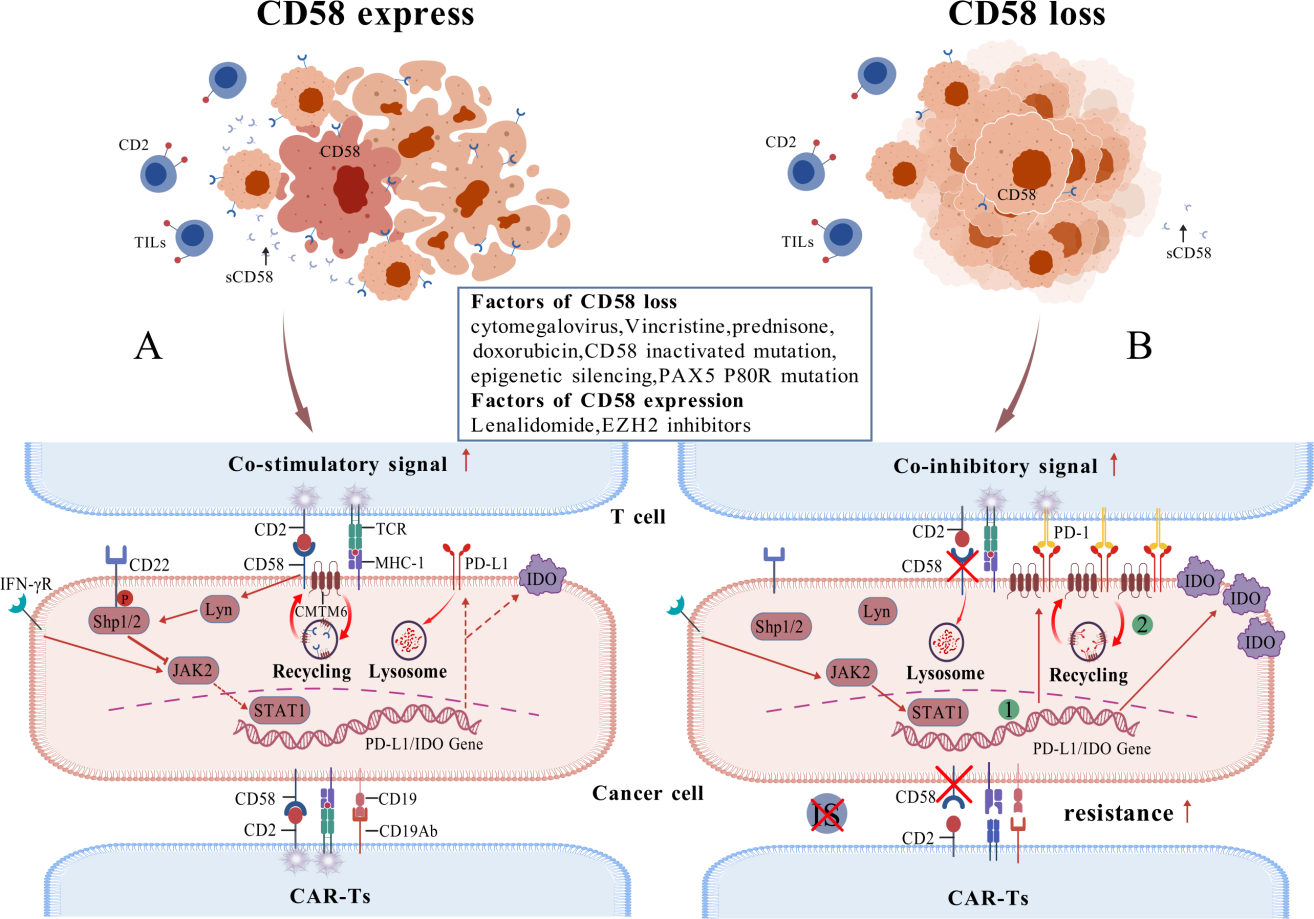
Relationship between CD58 and PD-L1 **(A)** illustrates a case of CD58 expression, where tumor cells exhibit diminished PD-L1 expression, leading to the activation of TIL and subsequent anti-tumor effects, while the tumor cells remain susceptible to CAR-T cell therapy; **(B)** portrays the case of CD58 loss, characterized by a substantial up-regulation of PD-L1 expression in tumor cells, resulting in ineffective TIL activation and evasion of cytotoxicity, alongside an evident resistance of tumor cells to CAR-T cell therapy. The green markers denote two mechanisms for upregulating PD-L1 expression: JAK/STAT pathway activation, increased binding of CMTM6 regulatory protein to PD-L1.).

Defective and diminished expression of the CD58 gene is significantly associated with lower response rates to R-CHOP (Chemotherapy regimens containing Rituximab, Cyclophosphamide, Doxorubicin, Vincristine, Prednisone) therapy and reduced PFS and OS (62, 66). Additionally, CD58 is among the most frequently mutated genes in relapsed or refractory DLBCL (67). Malladi et al. indicated that 20% of DLBCL cases exhibit genetic impairment of the single or double allele CD58 gene, and in cases devoid of CD58 gene defects, over half demonstrate undetectable CD58 protein expression via immunohistochemistry, resulting in diminished tumor cell recognition by Cytotoxic T Lymphocyte and NK cells (68). The epigenetic silencing of CD58 may correlate with EZH2, and applying EZH2 inhibitors promotes its demethylation and activates the transcription of the CD58 gene (69). Nevertheless, certain studies involving the Chinese population indicate that the mutation rate of CD58 is approximately 5%-10% (12), implying that the expression of CD58 may exhibit ethnic variation. Younes et al. propose that CD58 downregulation correlates with subtypes of DLBCL, indicating that the majority of newly diagnosed DLBCL CD58-positive patients do not belong to the Double-Hit/Triple-Hit subtype, while new-onset cases with a high International Prognostic Index (IPI) are inclined to have CD58 downregulation. Patients whose condition worsened following CAR-T cell therapy exhibited an increased incidence of CD58 aberrations (62). Majzner et al. reported that 24% of patients treated with Axicabtagene ciloleucel (Axi-cel) demonstrated impaired CD58 expression, correlating with a significant decrease in the durability of the Axi-cel response (70). Conversely, prior to CD19^+^CAR-T cell therapy, elevated CD58 expression in tumor samples was linked to improved clinical outcomes and survival rates (71). In conclusion, impairments in CD58 expression are typically linked to poorer prognosis, immune evasion, and drug resistance. Mechanistically, CD58 protein can indirectly inhibit PD-L1 expression in tumor cells through the following pathway: CD58 protein interacts with and activates Lyn kinase. Activated Lyn kinase then phosphorylates the immunoreceptor tyrosine inhibitory motif (ITIM) of CD22. The phosphorylated ITIM recruits and binds to protein tyrosine phosphatase 1 (SHP1), which contains an SH2 structural domain. This binding inhibits JAK2/STAT1 activity via the LYN/CD22/SHP1 signaling pathway, thereby suppressing the expression of PD-L1 and Indoleamine 2,3-dioxygenase (IDO) protein (72). Therefore, the loss of CD58 expression significantly increases PD-L1 and IDO protein expression. Additionally, Chemokine-like factor superfamily member 6 (CMTM6), which functions as a co-regulator of CD58 and PD-L1, directly promotes PD-L1 overexpression in the absence of CD58. Specifically, when tumor cells highly express CD58, CMTM6 binds to CD58 and promotes its stability via endosomal recycling, thereby inhibiting PD-L1 protein expression. Conversely, when CD58 is lost in tumor cells, CMTM6 switches to promoting PD-L1 expression (73–75). When PD-L1 is highly expressed, it interacts with PD-1 on T cells to block both TCR/MHC primary signaling and CD2/CD28 costimulatory signaling, thereby promoting immune evasion, and not associated with loss of MHC class I expression, IFN γ-JAK/STAT pathway (8). Second, CD58 is a critical component of the IS. When tumor cells lose CD58 expression, they become resistant to tumor-infiltrating lymphocytes (TIL), thereby further promoting immune evasion (**Figure 3**).

#### 4.2.3 Other subtypes of lymphoma

Research on systemic and cutaneous Dual Specificity Phosphatase-22 (DUSP-22) rearrangements in Anaplastic Lymphoma Kinase (ALK)-negative Anaplastic Large Cell Lymphoma (ALCL) has demonstrated positive CD58 expression, correlating with immune identification of the tumor and a favorable prognosis (76). Research by Younes et al. revealed that over 80% of ALCL cases had downregulation of CD58, with these cases also demonstrating ALK deficiency, whereas 75% of DUSP-22 positive individuals displayed positive CD58 expression (62). Teresa et al. also discovered CD58 mutations in peripheral T-cell lymphomas (77).The deletion of CD58 protein frequently occurs in adult T-cell leukemia/lymphoma (ATLL), furthermore, inactivating mutations in CD58 correlate with the aggressiveness of ATLL and the acute transformation of chronic-type ATLL (78). The expression of CD58 is markedly elevated in Burkitt’s Lymphoma (BL) compared to BCP-ALL, and it can be used to aid in the diagnosis when BL is suspected but MYC proto-oncogene (C-MYC) rearrangement is negative and the results of surface Ig assessment are controversial (79). A study involving 748 cases of various lymphoma subtypes revealed that CD58 protein expression was downregulated across all B-cell, T-cell, and NK-cell lymphomas; specifically, CD58 downregulation was observed in 14/102 (13.75%) cases of DLBCL, 40/84 (47.6%) cases of small B-cell lymphomas, and 24/29 (82.7%) cases of Follicular Lymphoma. Additionally, downregulation was noted in 53/69 (76.8%) cases of Mantle Cell Lymphoma and 39/48 (81.3%) cases of marginal zone lymphoma (62).

### 4.3 Multiple myeloma (MM)

One research on regulating MM antigen-specific T cell spatial architecture indicated that bone marrow-derived T cells from MM patients necessitated activation to infiltrate MM cell clusters. The extent of activation correlated with the type and intensity of the stimulus. Pretreatment of U266 MM cells with a CD 58 blocking antibody obstructed the CD2-CD58 interaction, thereby preventing activated T cells from entering the MM cluster (80). Malignant circulating plasma cells (MMCPCs) in peripheral blood (PB) serve as an independent prognostic indicator of rapid decline and markedly diminished overall survival in MM patients. It was found that the downregulation of the adhesive receptor CD58 is implicated in the mechanism by which malignant plasma cells (MMPCs) exit the bone marrow and infiltrate the PB, transforming into MMCPCs (81). These two studies suggest that CD58 is also closely related to the tumor immune response in MM.

## 5 Influence of CD58 on immunotherapy

### 5.1 Elevated CD58 expression enhances the effectiveness of CAR-T cell therapy

CAR-T cell therapy have demonstrated considerable advantages and potential applications in treating relapsed/refractory hematologic malignancies, including DLBCL and ALL (82). Research indicates that the IS established by the cooperation between CD58 of tumor cells and CD2 with CAR-T cells is essential for enhancing the cytotoxic efficacy of CAR-T cells (12, 83, 84). Additionally, the CD2-CD58 interaction is a significant adhesion axis that amplifies CAR signaling sensitivity, although the primary axis for enhancing CAR signaling is LFA-1/ICAM-1 (74). The downregulation of CD 58 in tumor cells impedes the establishment of IS with CAR-T cells (including CD19 CAR-T cells, CD20 CAR-T cells, and tandem CD19/20 CAR-T cells), leading to diminished CAR cell signaling, reduced cell growth, and impaired cytotoxicity, hence contributing to resistance against CAR-T cell therapy (12) (See **Figure 3**). Diverse strategies aimed at CD58 can augment sensitivity or mitigate resistance to CAR-T cell therapy. For cases structural domains alongside CAR molecules can circumvent the loss of CD58. in point, innovative CAR-T cells incorporating CD2 co-stimulatory Additionally, EZH2 inhibitors can reactivate epigenetically silenced CD58 expression, thereby enhancing clinical response. Consequently, CD58 may be a clinically predictive biomarker for evaluating responsiveness to CAR-T cell therapy.

### 5.2 Silencing CD58 diminishes immune rejection of CAR products

The silencing of β-2 micro-globulin, HLA class II transactivator gene expression, and other techniques for eradicating MHC-I and MHC-II protein can circumvent host CD8^+^ and CD4^+^ T cell-mediated rejection responses; however, this also renders donor cells vulnerable to loss of self-recognition by host NK cells, instigating NK cell-mediated immune rejection responses (46, 85). Current techniques that aim at HLA-E and CD47 are limited to facilitating partial NK cell-mediated immunological rejection, necessitating the urgent development of straightforward and effective novel ways (86, 87). CD54 and CD58 knockout CAR-T cells were observed to generally suppress host NK cell activity and exhibit relative resistance to NK cell assault; furthermore, CD54^-^/CD58^-^/B2M^-^ allogeneic CAR-T cells demonstrated improved survival compared to standard baseline CAR-T cells. Additionally, multiply edited CD54^-^/CD58^-^/iPSC^-^ derived CAR-NK cells displayed resistance to NK cell rejection *in vitro* and *in vivo* (46). Meanwhile, we have also considered the following questions: First, does knockdown of CD58 attenuate TCR signaling in CAR-T cells? (As previously mentioned, the cis interaction between CD2 and CD58 enhances TCR signaling). Second, could novel CAR constructs that integrate CD2 costimulatory domains while knocking out CD58 and/or CD54 surface molecules perform the dual functions of evading immune rejection and killing tumor cells that lack CD58 expression? Moreover, the existing methodologies for evaluating CAR products do not often accurately forecast the effectiveness of CAR-modified immune cells *in vivo*. Investigations are underway regarding strategies that utilize CAR-T or CAR-NK cell IS quality to anticipate the anti-tumor activity of these modified immune cells (83). Therefore, the significance of CD58, a crucial component of the IS, warrants emphasis.

### 5.3 The loss of CD58 contributes to bispecific T-cell engager resistance

BiTE molecules facilitate the redirected lysis of cancer cells by T cells, representing a novel approach in the immunotherapy of hematological malignancies, such as ALL (88). The absence of CD58 BiTE or the loss of interaction between CD2 and CD58 results in diminished TCE-β-mediated cytotoxicity, T cells activation, and anti-tumor efficacy, constituting a primary mechanism of BiTE resistance. Furthermore, the enhancement of CD2-CD58 axis signaling is significantly advantageous for BiTE treatment of tumors and assists in restoring the anti-tumor immunity of T cells (89).

In summary, CD58 expression in hematological tumors is frequently related to treatment response and longer survival, while CD58 loss contributes to immune evasion, facilitates the emergence of resistance to immunotherapies like CAR-T cell therapy, and indicates a poorer prognosis. The network of mechanisms regulating CD58 expression remains to be thoroughly studied; however, it is evident that doxorubicin, vincristine, and prednisone down-regulate CD58 expression, and cytotoxic drugs such as cyclophosphamide also inhibit CD58 expression in a dose-dependent manner (60). The immunomodulatory drug lenalidomide or the epigenetic modulator EZH2 inhibitor (EPZ6438, GSK126) have been observed to restore CD58 expression in cancer cells (69, 90). Future research should prioritize the development of therapeutic strategies, such as CD2 agonists, to restore CD58 expression in hematological tumors or to circumvent the CD2-CD58 axis to target CD58^-^ immuno-resistant cancer cells. Furthermore, the mechanism of expression regulation between CD58 and PD-L1 revealed that for CD58^-^PD-L1^+^ tumor cells, the strategy of co-targeting CD58 with PD-1/PD-L1 blockers may be more beneficial to activate T-cell killing, thus overcoming drug resistance in this tumor type. Finally, the therapeutic potential of Alefacept (a fusion protein consisting of the extracellular region of CD58 and the IgG1-Fc segment) in grafts against immune rejection has been explored (91), Studies of peptide (92, 93) and non-peptide antagonists (7) that target the extracellular structural domain of CD58 are also in progress. Therefore, CD58 antagonists maybe emerge as a potential strategy to reduce immune rejection of CAR products.

## 6 Conclusion

CD58 is a glycoprotein receptor that interacts with CD2 and is extensively present in immune cells and many tissue cell types. It is not only an essential molecule that forms the IS and activates T/NK cells but also plays a role in the immune response to malignancies. The absence of CD58 correlates with immune evasion and pharmacological resistance in hematological malignancies, including leukemia and lymphoma, and is a unique biomarker for forecasting clinical outcomes in the new era of immunotherapy.

## References

[1] Zeng H Yu J Wang H Shen M Zou X Zhang Z. Cancer ATF4-mediated CD58 endocytosis impairs anti-tumor immunity and immunotherapy. J Transl Med. (2025) 23:225. doi: 10.1186/s12967-025-06245-4 40001116 PMC11863482

[2] Jo Y Sim HI Yun B Park Y Jin HS. Revisiting T-cell adhesion molecules as potential targets for cancer immunotherapy: CD226 and CD2. Exp Mol Med. (2024) 56:2113–26. doi: 10.1038/s12276-024-01317-9 PMC11541569 39349829

[3] Wu B Zhan X Jiang M. CD58 defines regulatory macrophages within the tumor microenvironment. Commun Biol. (2024) 7:1025. doi: 10.1038/s42003-024-06712-6 39164573 PMC11335740

[4] Tian Z Jia W Wang Z Mao H Zhang J Shi Q. Clinical significance of immune-related antigen CD58 in gliomas and analysis of its potential core related gene clusters. Heliyon. (2024) 10:e29275. doi: 10.1016/j.heliyon.2024.e29275 38699747 PMC11063413

[5] Wu M Chen Y Hua G Chunhui L. The CD2-CD58 axis: A novel marker predicting poor prognosis in patients with low-grade gliomas and potential therapeutic approaches. Immun Inflammation Dis. (2023) 11:e1022. doi: 10.1002/iid3.1022 PMC10571499 37904707

[6] Wang C Cao F Cao J Jiao Z You Y Xiong Y. CD58 acts as a tumor promotor in hepatocellular carcinoma via activating the AKT/GSK-3β/β-catenin pathway. J Transl Med. (2023) 21:539. doi: 10.1186/s12967-023-04364-4 37573318 PMC10422835

[7] Guo R Yu J Guo Z. Virtual screening and binding analysis of potential CD58 inhibitors in colorectal cancer (CRC). Molecules. (2023) 28:6819. doi: 10.3390/molecules28196819 37836662 PMC10574072

[8] Frangieh CJ Melms JC Thakore PI Geiger-Schuller KR Ho P Luoma AM. Multimodal pooled Perturb-CITE-seq screens in patient models define mechanisms of cancer immune evasion. Nat Genet. (2021) 53:332–41. doi: 10.1038/s41588-021-00779-1 PMC8376399 33649592

[9] Melms JC Ho P Rogava M Izar B. From patient tissue correlates to molecular mechanisms of cancer immune evasion: the emerging role of CD58 and PD-L1 co-regulation via CMTM6. Genes Immun. (2024) 25:82–4. doi: 10.1038/s41435-023-00224-9 38082156

[10] Xiong Y Motomura H Tamori S Ozaki A Onaga C Hara Y. High expression of CD58 and ALDH1A3 predicts a poor prognosis in basal-like breast cancer. Anticancer Res. (2022) 42:5223–32. doi: 10.21873/anticanres.16029 36288878

[11] Zhang Y Liu Q Yang S Liao Q. CD58 immunobiology at a glance. Front Immunol. (2021) 12:705260. doi: 10.3389/fimmu.2021.705260 34168659 PMC8218816

[12] Yan X Chen D Ma X Wang Y Guo Y Wei J. CD58 loss in tumor cells confers functional impairment of CAR T cells. Blood Adv. (2022) 6:5844–56. doi: 10.1182/bloodadvances.2022007891 PMC9649996 35728062

[13] Liu F Romee R. A one-way street recognition approach to mediate allogeneic immune cell therapies. Cell Stem Cell. (2024) 31:1239–40. doi: 10.1016/j.stem.2024.07.005 39241752

[14] Ariel O Levi Y Hollander N. Signal transduction by CD58: the transmembrane isoform transmits signals outside lipid rafts independently of the GPI-anchored isoform. Cell Signal. (2009) 21:1100–8. doi: 10.1016/j.cellsig.2009.02.022 19268704

[15] Binder C Cvetkovski F Sellberg F Berg S Paternina Visbal H Sachs DH. CD2 immunobiology. Front Immunol. (2020) 11:1090. doi: 10.3389/fimmu.2020.01090 32582179 PMC7295915

[16] Itzhaky D Raz N Hollander N. The glycosylphosphatidylinositol-anchored form and the transmembrane form of CD58 are released from the cell surface upon antibody binding. Cell Immunol. (1998) 187:151–7. doi: 10.1006/cimm.1998.1323 9732704

[17] Scheibenbogen C Keilholz U Meuer S Dengler T Tilgen W Hunstein W. Differential expression and release of LFA-3 and ICAM-1 in human melanoma cell lines. Int J Cancer. (1993) 54:494–8. doi: 10.1002/ijc.2910540323 7685328

[18] Dustin ML. The immunological synapse. Cancer Immunol Res. (2014) 2:1023–33. doi: 10.1158/2326-6066.CIR-14-0161 PMC4692051 25367977

[19] Capera J Jainarayanan A Navarro-Pérez M Valvo S Demetriou P Depoil D. Dynamics and spatial organization of Kv1.3 at the immunological synapse of human CD4+ T cells. Biophys J. (2024) 123:2271–81. doi: 10.1016/j.bpj.2023.08.011 PMC11331042 37596785

[20] Demetriou P Abu-Shah E Valvo S McCuaig S Mayya V Kvalvaag A. A dynamic CD2-rich compartment at the outer edge of the immunological synapse boosts and integrates signals. Nat Immunol. (2020) 21:1232–43. doi: 10.1038/s41590-020-0770-x PMC7611174 32929275

[21] Liu J Chow VTK. Jois SDS. A novel, rapid and sensitive heterotypic cell adhesion assay for CD2-CD58 interaction, and its application for testing inhibitory peptides. J Immunol Methods. (2004) 291:39–49. doi: 10.1016/j.jim.2004.04.026 15345303

[22] Leitner J Herndler-Brandstetter D Zlabinger GJ Grubeck-Loebenstein B Steinberger P. CD58/CD2 is the primary costimulatory pathway in human CD28-CD8+ T cells. J Immunol. (2015) 195:477–87. doi: 10.4049/jimmunol.1401917 26041540

[23] Winchester NE Panigrahi S Haria A Chakraborty A Su X Chen B. Cytomegalovirus infection facilitates the costimulation of CD57+CD28- CD8 T cells in HIV infection and atherosclerosis via the CD2-LFA-3 axis. J Immunol. (2024) 212:245–57. doi: 10.4049/jimmunol.2300267 PMC10843654 38047900

[24] Chen B Morris SR Panigrahi S Michaelson GM Wyrick JM Komissarov AA. Cytomegalovirus coinfection is associated with increased vascular-homing CD57+ CD4 T cells in HIV infection. J Immunol. (2020) 204:2722–33. doi: 10.4049/jimmunol.1900734 PMC7315224 32229536

[25] Orlik C Berschneider KM Jahraus B Niesler B Balta E Schäkel K. Keratinocyte-induced costimulation of human T cells through CD6 - but not CD2 - activates mTOR and prevents oxidative stress. Front Immunol. (2022) 13:1016112. doi: 10.3389/fimmu.2022.1016112 36353616 PMC9639098

[26] Orlik C Deibel D Küblbeck J Balta E Ganskih S Habicht J. Keratinocytes costimulate naive human T cells via CD2: a potential target to prevent the development of proinflammatory Th1 cells in the skin. Cell Mol Immunol. (2020) 17:380–94. doi: 10.1038/s41423-019-0261-x PMC7109061 31324882

[27] Correa K Dustin ML. Locked and loaded: strong TCR signaling primes anti-PD-1 therapy. Trends Immunol. (2021) 42:1066–8. doi: 10.1016/j.it.2021.10.014 34772621

[28] Cvetkovski F Razavi R Sellberg F Berglund E Berglund D. Siplizumab combination therapy with belatacept or abatacept broadly inhibits human T cell alloreactivity. vitro. Am J Transplant. (2023) 23:1603–11. doi: 10.1016/j.ajt.2023.05.032 37270108

[29] Veldman J Visser L Huberts-Kregel M Muller N Hepkema B van den Berg A. Rosetting T cells in Hodgkin lymphoma are activated by immunological synapse components HLA class II and CD58. Blood. (2020) 136:2437–41. doi: 10.1182/blood.2020005546 PMC7685209 32589698

[30] Huppa JB Schütz GJ. T-cell antigen recognition: catch-as-catch-can or catch-22? EMBO J. (2023) 42:e113507. doi: 10.15252/embj.2023113507 36808636 PMC10068317

[31] Li B Lu Y Zhong MC Qian J Li R Davidson D. Cis interactions between CD2 and its ligands on T cells are required for T cell activation. Sci Immunol. (2022) 7:eabn6373. doi: 10.1126/sciimmunol.abn6373 35930657

[32] Db D Jp S Mr B Re R Gr C. Characterization of antigen receptor response elements within the interleukin-2 enhancer. Mol Cell Biol. (1988) 8(4):1715–24. doi: 10.1128/mcb.8.4.1715-1724.1988 PMC363332 3260003

[33] Parra E Varga M Hedlund G Kalland T Dohlsten M. Costimulation by B7–1 and LFA-3 targets distinct nuclear factors that bind to the interleukin-2 promoter: B7–1 negatively regulates LFA-3-induced NF-AT DNA binding. Mol Cell Biol. (1997) 17:1314–23. doi: 10.1128/MCB.17.3.1314 PMC231856 9032258

[34] Daniel PT Scholz C Essmann F Westermann J Pezzutto A Dörken B. CD95/Fas-triggered apoptosis of activated T lymphocytes is prevented by dendritic cells through a CD58-dependent mechanism. Exp Hematol. (1999) 27(9):1402–8. doi: 10.1016/s0301-472x(99)00079-x 10480431

[35] Lopez RD Waller EK Lu PH Negrin RS. CD58/LFA-3 and IL-12 provided by activated monocytes are critical in the *in vitro* expansion of CD56+ T cells. Cancer immunology immunotherapy: CII. (2001) 49:629–40. doi: 10.1007/s002620000148 PMC11036977 11258789

[36] Kristenson L Badami C Ljungberg A Islamagic E Tian Y Xie G. Deletion of the TMEM30A gene enables leukemic cell evasion of NK cell cytotoxicity. Proc Natl Acad Sci U S A. (2024) 121:e2316447121. doi: 10.1073/pnas.2316447121 38557174 PMC11009675

[37] Grier JT Forbes LR Monaco-Shawver L Oshinsky J Atkinson TP Moody C. Human immunodeficiency-causing mutation defines CD16 in spontaneous NK cell cytotoxicity. J Clin Invest. (2012) 122:3769–80. doi: 10.1172/JCI64837 PMC3461929 23006327

[38] Pech MF Fong LE Villalta JE Chan LJ Kharbanda S O'Brien JJ. Systematic identification of cancer cell vulnerabilities to natural killer cell-mediated immune surveillance. eLife. (2019) 8:e47362. doi: 10.7554/eLife.47362 31452512 PMC6713475

[39] Schwane V Huynh-Tran VH Vollmers S Yakup VM Sauter J Schmidt AH. Distinct signatures in the receptor repertoire discriminate CD56bright and CD56dim natural killer cells. Front Immunol. (2020) 11:568927. doi: 10.3389/fimmu.2020.568927 33335526 PMC7736243

[40] Ross R Hasheminasab SS Conejeros I Gärtner U Kamena F Krueger A. Human dendritic cell interactions with the zoonotic parasite Cryptosporidium parvum result in activation and maturation. Front Immunol. (2024) 15:1388366. doi: 10.3389/fimmu.2024.1388366 38799470 PMC11116633

[41] Nguyen KG Vrabel MR Mantooth SM Hopkins JJ Wagner ES Gabaldon TA. Localized interleukin-12 for cancer immunotherapy. Front Immunol. (2020) 11:575597. doi: 10.3389/fimmu.2020.575597 33178203 PMC7593768

[42] Gollob JA Li J Reinherz EL Ritz J. CD2 regulates responsiveness of activated T cells to interleukin 12. J Exp Med. (1995) 182:721–31. doi: 10.1084/jem.182.3.721 PMC2192171 7544396

[43] Gollob JA Li J Kawasaki H Daley JF Groves C Reinherz EL. Molecular interaction between CD58 and CD2 counter-receptors mediates the ability of monocytes to augment T cell activation by IL-12. J Immunol (Baltimore Md: 1950). (1996) 157:1886–93. 8757306

[44] Yamamoto M Watanabe M Inoue N Watanabe A Ozaki H Ohsaki M. Association of CD58 polymorphisms and its protein expression with the development and prognosis of autoimmune thyroid diseases. Immunol Invest. (2020) 49:106–19. doi: 10.1080/08820139.2019.1659811 31505972

[45] Boris E Theron A Montagnon V Rouquier N Almeras M Moreaux J. Immunophenotypic portrait of leukemia-associated-phenotype markers in B acute lymphoblastic leukemia. Cytometry B Clin Cytom. (2024) 106:45–57. doi: 10.1002/cyto.b.22153 38037221

[46] Hammer Q Perica K Mbofung RM van Ooijen H Martin KE Momayyezi P. Genetic ablation of adhesion ligands mitigates rejection of allogeneic cellular immunotherapies. Cell Stem Cell. (2024) 31:1376–1386.e8. doi: 10.1016/j.stem.2024.06.011 38981470 PMC12718542

[47] Manes TD Wang V Pober JS. Costimulators expressed on human endothelial cells modulate antigen-dependent recruitment of circulating T lymphocytes. Front Immunol. (2022) 13:1016361. doi: 10.3389/fimmu.2022.1016361 36275645 PMC9582530

[48] Berner J Weiss T Sorger H Rifatbegovic F Kauer M Windhager R. Human repair-related Schwann cells adopt functions of antigen-presenting cells. vitro. Glia. (2022) 70:2361–77. doi: 10.1002/glia.24257 PMC9804420 36054432

[49] Shi G Yang C Zhou L Zong M Guan Q da Roza G. Comprehensive cell surface protein profiling of human mesenchymal stromal cells from peritoneal dialysis effluent and comparison with those from human bone marrow and adipose tissue. Hum Cell. (2023) 36:2259–69. doi: 10.1007/s13577-023-00971-x PMC10587256 37603218

[50] Wolfmeier H Heindl S Platzl C Kaser-Eichberger A Nematian-Ardestani E Strohmaier C. Targeted surface marker screening on neuronal structures in the human choroid. Exp Eye Res. (2023) 227:109368. doi: 10.1016/j.exer.2022.109368 36586549

[51] Pierzyna-Świtała M Sędek Ł Kulis J Mazur B Muszyńska-Rosłan K Kołtan A. Multicolor flow cytometry immunophenotyping and characterization of aneuploidy in pediatric B-cell precursor acute lymphoblastic leukemia. Cent Eur J Immunol. (2021) 46:365–74. doi: 10.5114/ceji.2021.109794 PMC8574114 34764809

[52] Verbeek MWC van der Velden VHJ. The evolving landscape of flowcytometric minimal residual disease monitoring in B-cell precursor acute lymphoblastic leukemia. Int J Mol Sci. (2024) 25:4881. doi: 10.3390/ijms25094881 38732101 PMC11084622

[53] Gupta S Devidas M Loh ML Raetz EA Chen S Wang C. Flow-cytometric vs. -morphologic assessment of remission in childhood acute lymphoblastic leukemia: a report from the Children’s Oncology Group (COG). Leukemia. (2018) 32:1370–9. doi: 10.1038/s41375-018-0039-7 PMC5992047 29472723

[54] Thulasi Raman R Anurekha M Lakshman V Balasubramaniam R Ramya U Revathi R. Immunophenotypic modulation in pediatric B lymphoblastic leukemia and its implications in MRD detection. Leuk Lymphoma. (2020) 61:1974–80. doi: 10.1080/10428194.2020.1742902 32281503

[55] Diamanti P Cox CV Ede BC Uger RA Moppett JP Blair A. Targeting pediatric leukemia-propagating cells with anti-CD200 antibody therapy. Blood Adv. (2021) 5:3694–708. doi: 10.1182/bloodadvances.2020003534 PMC8945591 34470052

[56] Davydova Y Galtseva I Kapranov N Nikiforova K Aleshina O Chabaeva Y. Immunophenotype of measurable residual blast cells as an additional prognostic factor in adults with B-cell acute lymphoblastic leukemia. Diagnostics (Basel). (2022) 13:21. doi: 10.3390/diagnostics13010021 36611312 PMC9818326

[57] Bisegna ML Peragine N Elia L Matarazzo M Milani ML Intoppa S. NG2 molecule expression in acute lymphoblastic leukemia B cells: A flow-cytometric marker for the rapid identification of KMT2A gene rearrangements. Mediterr J Hematol Infect Dis. (2024) 16:e2024018. doi: 10.4084/MJHID.2024.018 38468826 PMC10927233

[58] Li Y Moriyama T Yoshimura S Zhao X Li Z Yang X. PAX5 epigenetically orchestrates CD58 transcription and modulates blinatumomab response in acute lymphoblastic leukemia. Sci Adv. (2022) 8:eadd6403. 36516256 10.1126/sciadv.add6403 PMC9750140

[59] Arumugam JR Bommannan K Radhakrishnan V Sagar TG Sundersingh S. Immunophenotypic expression and immunomodulation in minimal residual disease analysis of pediatric B acute lymphoblastic leukemia by high sensitive flow cytometry. Leuk Lymphoma. (2022) 63:644–52. doi: 10.1080/10428194.2021.1992755 34727819

[60] Prelog T Bucek S Brozic A Peterlin J Kavcic M Omerzel M. The influence of cytotoxic drugs on the immunophenotype of blast cells in paediatric B precursor acute lymphoblastic leukaemia. Radiol Oncol. (2024) 58:133–44. doi: 10.2478/raon-2024-0006 PMC10878768 38378030

[61] Chen C Chen Z Chio CL Zhao Y Li Y Liu Z. Higher Expression of WT1 With Lower CD58 Expression may be Biomarkers for Risk Stratification of Patients With Cytogenetically Normal Acute Myeloid Leukemia. Technol Cancer Res Treat. (2021) 20:15330338211052152. doi: 10.1177/15330338211052152 34738847 PMC8573474

[62] Younes S Zhao S Bharadwaj S Mosquera AP Libert D Johnsrud A. Detection of aberrant CD58 expression in a wide spectrum of lymphoma subtypes: implications for treatment resistance. Mod Pathol. (2023) 36:100256. doi: 10.1016/j.modpat.2023.100256 37391168

[63] Schneider M Schneider S Zühlke-Jenisch R Klapper W Sundström C Hartmann S. Alterations of the CD58 gene in classical Hodgkin lymphoma. Genes Chromosomes Cancer. (2015) 54:638–45. doi: 10.1002/gcc.22276 26194173

[64] Casagrande N Borghese C Avanzo M Aldinucci D. In doxorubicin-adapted hodgkin lymphoma cells, acquiring multidrug resistance and improved immunosuppressive abilities, doxorubicin activity was enhanced by chloroquine and GW4869. Cells. (2023) 12:2732. doi: 10.3390/cells12232732 38067159 PMC10706762

[65] Cader FZ Schackmann RCJ Hu X Wienand K Redd R Chapuy B. Mass cytometry of Hodgkin lymphoma reveals a CD4+ regulatory T-cell-rich and exhausted T-effector microenvironment. Blood. (2018) 132:825–36. doi: 10.1182/blood-2018-04-843714 PMC6107878 29880615

[66] Pedrosa L Fernández-Miranda I Pérez-Callejo D Quero C Rodríguez M Martín-Acosta P. Proposal and validation of a method to classify genetic subtypes of diffuse large B cell lymphoma. Sci Rep. (2021) 11:1886. doi: 10.1038/s41598-020-80376-0 33479306 PMC7820010

[67] Rushton CK Arthur SE Alcaide M Cheung M Jiang A Coyle KM. Genetic and evolutionary patterns of treatment resistance in relapsed B-cell lymphoma. Blood Advances. (2020) 4:2886–98. doi: 10.1182/bloodadvances.2020001696 PMC7362366 32589730

[68] Challa-Malladi M Lieu YK Califano O Holmes AB Bhagat G Murty VV. Combined genetic inactivation of β2-Microglobulin and CD58 reveals frequent escape from immune recognition in diffuse large B cell lymphoma. Cancer Cell. (2011) 20:728–40. doi: 10.1016/j.ccr.2011.11.006 PMC3660995 22137796

[69] Otsuka Y Nishikori M Arima H Izumi K Kitawaki T Hishizawa M. EZH2 inhibitors restore epigenetically silenced CD58 expression in B-cell lymphomas. Mol Immunol. (2020) 119:35–45. doi: 10.1016/j.molimm.2020.01.006 31962268

[70] Majzner RG Frank MJ Mount C Tousley A Mackall CL. CD58 Aberrations Limit Durable Responses to CD19 CAR in Large B Cell Lymphoma Patients Treated with Axicabtagene Ciloleucel but Can be Overcome through Novel CAR Engineering. Blood. (2020) 136:53–4. doi: 10.1182/blood-2020-139605

[71] Romain G Strati P Rezvan A Fathi M Bandey IN Adolacion JRT. Multidimensional single-cell analysis identifies a role for CD2-CD58 interactions in clinical antitumor T cell responses. J Clin Invest. (2022) 132:e159402. doi: 10.1172/JCI159402 35881486 PMC9433104

[72] Xu X Zhang Y Lu Y Zhang X Zhao C Wang J. CD58 alterations govern antitumor immune responses by inducing PDL1 and IDO in diffuse large B-cell lymphoma. Cancer Res. (2024) 84:2123–40. doi: 10.1158/0008-5472.CAN-23-2874 38635903

[73] Ho P Melms JC Rogava M Frangieh CJ Poźniak J Shah SB. The CD58-CD2 axis is co-regulated with PD-L1 via CMTM6 and shapes anti-tumor immunity. Cancer Cell. (2023) 41:1207–1221.e12. doi: 10.1016/j.ccell.2023.05.014 37327789 PMC10524902

[74] Patel A Andre V Eguiguren SB Barton MI Burton J Denham EM. Using CombiCells, a platform for titration and combinatorial display of cell surface ligands, to study T-cell antigen sensitivity modulation by accessory receptors. EMBO J. (2024) 43:132–50. doi: 10.1038/s44318-023-00012-1 PMC10897201 38177315

[75] Miao B Hu Z Mezzadra R Hoeijmakers L Fauster A Du S. CMTM6 shapes antitumor T cell response through modulating protein expression of CD58 and PD-L1. Cancer Cell. (2023) 41:1817–1828.e9. doi: 10.1016/j.ccell.2023.08.008 37683639 PMC11113010

[76] Ravindran A Feldman AL Ketterling RP Dasari S Rech KL McPhail ED. Striking association of lymphoid enhancing factor (LEF1) overexpression and DUSP22 rearrangements in anaplastic large cell lymphoma. Am J Surg Pathol. (2021) 45:550–7. doi: 10.1097/PAS.0000000000001614 33165091

[77] Palomero T Couronné L Khiabanian H Kim MY Ambesi-Impiombato A Perez-Garcia A. Recurrent mutations in epigenetic regulators, RHOA and FYN kinase in peripheral T cell lymphomas. Nat Genet. (2014) 46:166–70. doi: 10.1038/ng.2873 PMC3963408 24413734

[78] Yoshida N Karube K Utsunomiya A Tsukasaki K Imaizumi Y Taira N. Molecular characterization of chronic-type adult T-cell leukemia/lymphoma. Cancer Res. (2014) 74:6129–38. doi: 10.1158/0008-5472.CAN-14-0643 25320005

[79] Demina I Voropayev A Semchenkova A Zerkalenkova E Olshanskaya Y Samochatova E. Additional flow cytometric studies for differential diagnosis between Burkitt lymphoma/leukemia and B-cell precursor acute lymphoblastic leukemia. Leuk Res. (2021) 100:106491. doi: 10.1016/j.leukres.2020.106491 33340851

[80] Robinson MH Villa NY Jaye DL Nooka AK Duffy A McCachren SS. Regulation of antigen-specific T cell infiltration and spatial architecture in multiple myeloma and premalignancy. J Clin Invest. (2023) 133:e167629. doi: 10.1172/JCI167629 37526080 PMC10378152

[81] Klimienė I Radzevičius M Matuzevičienė R Sinkevič-Belliot K Kučinskienė ZA Pečeliūnas V. Adhesion molecule immunophenotype of bone marrow multiple myeloma plasma cells impacts the presence of Malignant circulating plasma cells in peripheral blood. Int J Lab Hematol. (2021) 43:403–8. doi: 10.1111/ijlh.13387 33185981

[82] Lesch S Benmebarek MR Cadilha BL Stoiber S Subklewe M Endres S. Determinants of response and resistance to CAR T cell therapy. Semin Cancer Biol. (2020) 65:80–90. doi: 10.1016/j.semcancer.2019.11.004 31705998

[83] Liu D Badeti S Dotti G Jiang JG Wang H Dermody J. The role of immunological synapse in predicting the efficacy of chimeric antigen receptor (CAR) immunotherapy. Cell communication signaling: CCS. (2020) 18:134. doi: 10.1186/s12964-020-00617-7 32843053 PMC7446110

[84] Xiong W Chen Y Kang X Chen Z Zheng P Hsu YH. Immunological synapse predicts effectiveness of chimeric antigen receptor cells. Mol Therapy: J Am Soc Gene Ther. (2021) 29:1349–51. doi: 10.1016/j.ymthe.2021.01.025 PMC7934788 33592166

[85] Hu X White K Olroyd AG DeJesus R Dominguez AA Dowdle WE. Hypoimmune induced pluripotent stem cells survive long term in fully immunocompetent, allogeneic rhesus macaques. Nat Biotechnol. (2024) 42:413–23. doi: 10.1038/s41587-023-01784-x PMC10940156 37156915

[86] Vivier E Rebuffet L Narni-Mancinelli E Cornen S Igarashi RY Fantin VR. Natural killer cell therapies. Nature. (8000) 2024:626. doi: 10.1038/s41586-023-06945-1 38383621

[87] Deuse T Hu X Agbor-Enoh S Jang MK Alawi M Saygi C. The SIRPα-CD47 immune checkpoint in NK cells. J Exp Med. (2021) 218::e20200839. doi: 10.1084/jem.20200839 33416832 PMC7802363

[88] Kantarjian H Stein A Gökbuget N Fielding AK Schuh AC Ribera JM. Blinatumomab versus chemotherapy for advanced acute lymphoblastic leukemia. N Engl J Med. (2017) 376:836–47. doi: 10.1056/NEJMoa1609783 PMC5881572 28249141

[89] Shen Y Eng JS Fajardo F Liang L Li C Collins P. Cancer cell-intrinsic resistance to BiTE therapy is mediated by loss of CD58 costimulation and modulation of the extrinsic apoptotic pathway. J Immunother Cancer. (2022) 10:e004348. doi: 10.1136/jitc-2021-004348 35296559 PMC8928392

[90] Gribben JG Fowler N Morschhauser F. Mechanisms of action of lenalidomide in B-cell non-hodgkin lymphoma. J Clin Oncology: Off J Am Soc Clin Oncology. (2015) 33:2803–11. doi: 10.1200/JCO.2014.59.5363 PMC5320950 26195701

[91] Kyriakidis I Vasileiou E Rossig C Roilides E Groll AH Tragiannidis A. Invasive fungal diseases in children with hematological Malignancies treated with therapies that target cell surface antigens: monoclonal antibodies, immune checkpoint inhibitors and CAR T-cell therapies. J Fungi (Basel Switzerland). (2021) 7:186. doi: 10.3390/jof7030186 PMC7999508 33807678

[92] Tripathi N Leherte L Vercauteren DP Laurent AD. Structure-based identification of inhibitors disrupting the CD2-CD58 interactions. J Comput Aided Mol Des. (2021) 35:337–53. doi: 10.1007/s10822-020-00369-z 33532888

[93] Parajuli P Sable R Shrestha L Dahal A Gauthier T Taneja V. Modulation of co-stimulatory signal from CD2-CD58 proteins by a grafted peptide. Chem Biol Drug Des. (2021) 97:607–27. doi: 10.1111/cbdd.13797 PMC8717467 32946175

